# Evolution of language assessment in patients with acquired neurological disorders in Brazil

**DOI:** 10.1590/S1980-57642014DN83000002

**Published:** 2014

**Authors:** Maria Alice de Mattos Pimenta Parente, Roberta Roque Baradel, Rochele Paz Fonseca, Natalie Pereira, Maria Teresa Carthery-Goulart

**Affiliations:** 1 Senior Professor (PVNS/ CAPES) at UFABC – Universidade Federal do ABC. Center of Mathematics, Computation and Cognition. Neuroscience and Cognition Post-graduate Program. Language and Cognition Research Group (GELC-UFABC), Santo André, SP, Brazil; 2 Post-graduate student (MSc). Neuroscience and Cognition Post-graduate Program, UFABC – Universidade Federal do ABC. Language and Cognition Research Group (GELC-UFABC), Santo André, SP, Brazil; 3 Adjunct Professor with the Psychology Department, Post-Graduate Program in Psychology, Human Cognition Pontifical Catholic University of Rio Grande do Sul (PUCRS); 4 Post-graduate student (MSc). Post-Graduate Program in Psychology, Human Cognition; Pontifical Catholic University of Rio Grande do Sul (PUCRS); 5 Professor at UFABC – Universidade Federal do ABC. Center of Mathematics, Computation and Cognition. Neuroscience and Cognition Post-graduate Program. Language and Cognition Research Group (GELC-UFABC), Santo André, SP, Brazil. Researcher at the Behavioural and Cognitive Neurology Unit, University of São Paulo, School of Medicine, São Paulo, Brazil.

**Keywords:** acquired aphasia, disability evaluation, history

## Abstract

The objective of this paper was to describe the evolution of language assessments in patients with acquired neurological diseases over a period of around 45 years from 1970, when interdisciplinarity in Neuropsychology first began in Brazil, to the present day. The first twenty years of data was based on memories of Speech Pathology University Professors who were in charge of teaching aphasia. We then show the contributions of Linguistics, Cognitive Psychology, as well as Psycholinguistic and Psychometric criteria, to language evaluation. Finally, the current panorama of adaptations and creations of validated and standardized instruments is given, based on a search of the databases Pubmed, Scopus and Lilacs. Our closing remarks highlight the diversity in evaluation approaches and the recent tendency of language evaluations linked to new technologies such as brain imaging and computational analysis.

## Abstract (pt)

O objetivo deste artigo é descrever a evolução das avaliações de linguagem em pacientes com distúrbios neurológicos adquiridos durante um período de aproximadamente 45 anos, dos anos 1970 até os dias atuais, quando a interdisciplinaridade iniciou-se no Brasil na Neuropsicologia. Os relatos dos primeiros 20 anos foram baseados em memórias de professores de universidades que possuiam o curso de Fonoaudiologia e ministravam disciplinas de afasia. Após, procuramos mostrar as contribuições da Linguística, da Psicologia Cognitiva para a avaliação da linguagem, assim como dos critérios psicolinguísticos e psicométricos. Por fim, apresentamos o panorama atual das adaptações e criações de instrumentos validados e estandardizados a partir de busca nas bases Pubmed, Scopus e Lilacs. Nossos comentários finais salientam a diversidade dos métodos de avaliação e a tendência recente da avaliação da linguagem estar relacionada às novas tecnologias de imagem cerebral e a análises computacionais.

## INTRODUCTION

An important development in the evaluation of acquired disorders of Language* in neurological patients occurred during the period of the World Wars, when interdisciplinarity took place. During this period, Language evaluation of brain patients, then restricted to medical practice, began to be influenced by Linguistics and Psychology. In Brazil, the first descriptions of aphasia are found in the beginning of the 20th century, with the works and thesis supervised by Prof. Antônio Austregésilo^1,2^ at the Medical Faculty of the Federal University of Rio de Janeiro. Instruments for Language assessments were already evident in the 1950s, in the publication "New test for diagnosis of aphasia",^3^ but aphasia evaluation remained a medical domain. The influence of speech therapists that worked during and after the World Wars, demonstrating the benefits of interdisciplinarity, occurred in Brazil only in the 1970s. The interaction between neurological, linguistic and psychological sciences marked several changes in the way of evaluating the Language of brain-damaged patients thereafter. Thus, the objective of this paper was to show the evolution of Language assessment in patients with acquired neurological diseases, during a period of around 45 years, from 1970 to the present day. Several publications during 1970-1990s were written in journals or books that are now out of print. Thus, it is a major challenge to tell the story of this initial period. In contrast to other reviews that can be built from keywords in internet searches, we needed to draw on the memories of Professors of Speech Pathology schools at that time. After the 1990s, approaches from Linguistics and Psychology contributed toward establishing scientific criteria in Language evaluation. These areas, with different methodological proposals have been improving Language assessment instruments, constructed according to current theories and disseminated in the scientific community by publications.

In order to show this evolution, the present paper is organized into the following sections:

(1) memories from 1970 to 1990;
(2) the contributions of Linguistics to Language evaluation;
(3) the Psychological contribution, i.e., the Psycholinguistic and Psychometric criteria in Language assessment; and finally
(4) the current panorama of adaptation and creation of validated and standardized instruments.

## MEMORIES: LANGUAGE ASSESSMENT (1970-1990)

In the period between 1970 and 1990 there were great changes in Neuropsychology that impacted Language assessment practices in Brazil. Computed Tomography was a recent development and the few services in the country offering this exam did not use it routinely. Thus, assessments played a role both in aiding the localization of lesions and for rehabilitation purposes.

The theory of aphasia was under construction. In the early 1970s we were influenced by the one-dimensional and multidimensional conceptions of aphasia. The first, by Schuell et al.^4^ and Jones and Wepman,^5^ attributed the diversity of aphasic types to the variation of the severity of a single episode. By contrast, the group vision by Borod, Goodglass and Caplan^6^ identified clinical pictures distinguished by the nature of the deficit according to a multidimensional perspective that also generated reflections on how to assess and treat aphasics. In both these perspectives, the metalinguistic view of Language prevailed.

Internationally, knowledge diffusely published on the topic began to take shape and in 1970 we had access to Martha Taylor Sarno's^7^ compilation of texts Selected Readings about Aphasia. This book featured a panel of models that provided support for assessment: psycholinguistic and heuristic (empirical) models and the first signs of concern with psychosocial models and functionality. Other sources of information at the time included the books by Luria^8^ and Russian neuropsychologists. For the first time we had theoretical guidelines that discussed, justified and advocated a set of practices.

The automatic-voluntary dissociation observed in aphasia justified the interpretation of language integrity and the attribution of the main problem to accessing linguistic knowledge. Thus, initially, clinical observation of behavior was the main source for selecting interventions and guidance for patients and families. There was great concern with the oscillations in the behavior of aphasics – Language comprehension and production –, and, of course, with the facilitating factors underlying the oscillations in performance, attributed to the social environment.

Among the instruments available in our milieu was Schuell's^4^ Minnesota test, translated and adapted to Portuguese in 1978 by Maria Isis Marinho Meira.^9^ To our knowledge, this adaptation was a landmark that triggered the discussion about the need to consider the cultural impact of tests produced in other languages. She recognized the need to modify the text evaluating oral comprehension due to its leanings towards American Anglo-Saxon but not Brazilian culture.

The Boston test,^10^ available in English in the 1980s, was also a reference for assessing aphasics, even though it was not validated for Portuguese according to psychometric standards. In this test, there is greater emphasis on verbal and nonverbal aspects, with grading of difficulties, as well as on the interaction of Language and other cognitive aspects, addressed by the calculus exam, visual-spatial aspects, gnosis and praxis. A notable feature was the content of the texts intended for oral comprehension, with references to American soldiers in camp and items entirely unrelated to Brazilian culture in the naming tests. The first aphasia batteries that used psycholinguistic criteria and adaptation of the stimuli to the structure of the Brazilian Portuguese language were most probably the Montreal-Toulouse test (Alpha Version) and a long version (Beta Version), although these instruments were not validated and standardized. The Alpha version was intended for bedside evaluation. The Beta version was created for use in research and evaluated patients' strategies as well as their errors and preserved capacities on several linguistics tasks, such as naming, repetition, oral and written comprehension of words and sentences, text reading and writing skills. In Rio de Janeiro, also influenced by the French/Canadian school, Regina Jacoboviks discussed the evaluation of aphasia in her book "Introdução às afasias".^11^

In the 1980s two important sets of ideas gained strength: those modeled on cognitive models and those based on social constructs for the assessment and rehabilitation of aphasia. We were influenced by both, particularly by the psychosocial models. These lines awoke us to the importance of a multidisciplinary approach in the construction of clinical practices of assessment and therapy directed to aphasics. Cognitive Psychology gave prominence to a cognitive construct on hypothetical basis, while the Pragmatic approach highlighted the social environment and functionality. Both were developed at a time when there was little information about the neurobiological substrate. The coexistence of these two lines reflects the range of views during the period: Cognitivism, concerned with the construction of hypotheses about how Language functions and formal rigor of case studies; Pragmatics, concerned with the individual in his environment and social parameters that were resistant to formalization.

It is important to mention the Aphasia Congress, organized by us (MAMPP) in São Paulo in June of 1980. Renowned Brazilian and International researchers, held debates on tools and theoretical and methodological issues related to Language assessment, among other activities. The discussion proposed by Yvan Lebrun^12^ on functional aspects in Language assessment as opposed to the metalinguistic view, hitherto predominant, strengthened both theoretical and practical interest and influenced several groups of Brazilians active in the fields of Neuropsychology and Neurolinguistics.

Among the instruments concerned with the functionality of aphasics, an important one was the CADL – Communication Abilities in Daily Living, developed by A. Holland.^13^ Its script moved away from the metalinguistic Language model, but created another bias by relying on the representation of an interaction and not on real communication. Thus, metalanguage was replaced by metacommunication. Our attempts to use this instrument ran into important socio-cultural barriers, especially when examining patients with restricted access to formal education.

The research on social factors that are determinant to the physiology of the human brain in 1981 brought us close to the screening test for Language Alpha, developed by Lecours et al.,^14,15^ which was aligned with the multidimensional view of aphasia. The most important aspect of this experience was to clarify the awareness of cultural factors in the assessment, in particular education and the importance of a global view of the patients and the construction of a Language database.

The proposed therapy for aphasia of the postwar period was unquestionable as a social compensation mechanism. However, the extension of the therapeutic practices to society required evidence. Thus, Language assessment sought convincing results that would justify rehabilitation. Vignolo's article^16^ showed the importance of assessment to verify the effectiveness of therapeutic interventions and discussion of the control group of aphasics (in this case those with no access to therapy).

In the assessment of aphasia, the metalinguistic view prevailed, in which the finding of therapeutic results was endorsed by test performance. The unfavorable view of therapy published by Lincoln^17^ had great practical impact at the time. The care service for aphasics closed down in São Paulo and skepticism about therapy led many neurologists to question the benefits of referral for Language rehabilitation. Fortunately this situation was reversed, driven by the beginning of discussions of the World Health Organization^18^ stressing the concept of health from the perspective of social inclusion and participation. At that time, the position of authorities such as Albert and Helm-Estabrooks^19^ also strengthened the view of rehabilitation within a comprehensive perspective of communication.

When examining these historical facts with the passage of time we see the richness and timeliness of the contributions made in the years spanning between 1970 and 1990. During this period, the multidisciplinary view of language assessment in aphasia widened, deepened and consolidated. This knowledge was the basis for the construction of instruments for the assessment of other Language changes such as dementia and also for the conception of Language assessment integrated with other cognitive aspects.

If we examine the publications on modern conceptions of Broca's area,^20^ we see the contemporaneity of Schuell's^4^ vision. Today, Broca's area is seen as responsible for the synthesis of verbal and nonverbal information and for processing them into articulate speech. On the other hand, cognitive models have also gained space today, validated and complemented by Neuroimaging in views of connected networks. Additionally, the development of constructs such as theory of mind, and the investigation of its substrate, attest to the importance of assessments that consider communicative acts and intentions of the speaker.

Summing up, the 1970-1990 period was extremely fertile and important for the development of Language assessment in Neuropsychology. Currently, Language assessment is based on many of the constructs built during this period and new models that integrate biological and social views attest to the importance of considering standardized measures that take into account cultural and ecological aspects.

## CONTRIBUTIONS OF LINGUISTICS TO LANGUAGE ASSESSMENT

Linguistics is the basis for Language evaluation and, researchers such Claudia Thereza Guimaraes de Lemos and Mary Kato, who were lecturers both at PUC-São Paulo and UNICAMP, had great influence in the education of those who studied between the 1970's and 1990s, as well as those who currently work in Language evaluation of patients with neurological disorders.

Modern Linguistics study chooses language as an object of study and, from this, is devoted to different theoretical approaches such as syntactic, phonological, semantic, discursive, historical and social bases.^21^ Thus, in Contemporary Linguistics, as new theoretical and technological possibilities arose, new theoretical paths needed to be plotted and the structuralist, behaviorist and evolutionist trends became more distant from current investigative needs. In turn, new organicist, cognitive and interactionist postulates emerged, thus allowing Linguistics to become what it is today: a multifaceted, multidisciplinary science that deals with the study of Language in its various aspects.

This new setting was an essential contributor to the field of Cognitive Neuropsychology, since after the cognitive revolution and the influence of interdisciplinary works - such as Alajouanine, Ombredane and Durand^22^ and psycholinguistics theories, which foresaw Language from intermediate levels of representation, such as Van Dijk and Kintsch^23^ - it was possible to strengthen the interdisciplinary dialogue not only with Cognitive Neuropsychology, but also with the field of Aphasiology and Language Assessment in the normal and pathological research context.

Accordingly, with the advances of Neuroscience, Language assessment, which until the 1980s was limited to observing only formal aspects related to mental and linguistic concepts, started to incorporate more detailed investigative techniques that allowed the analysis of not only the functioning of language from strategies that predicted structural and functional aspects, but also investigated, in a interrelated manner, other contextual mechanisms underlying the cognitive processes involved. These approaches can be divided into four main areas^24^:

1. The organicist-localizationist - affiliated to the notion of lesion-symptom, created and inspired by Broca and Wernicke - where there is a strong influence of the taxonomy and naturalistic thinking, focused on the description, identification and classification of language as a coded communication apparatus. In this theoretical framework, in the name of scientific rationality, emphasis is given to objective measurement of the functioning of Language specificities tied to cortical areas;
2. Neurolinguistic-structuralist - affiliated to the theories of the Russian Neuropsychologists, evaluating complex mental phenomena resulting from the activity of the entire brain, organized by functional systems. It is defined here that Language is one of the manifestations of these phenomena, a decisive tool and essential base of knowledge and human thought;
3. Enunciative-discursive, emerged in Brazil in the late 1980s, considering concepts and practices that articulate speech within contextual and constitutive activity of subjects in their individuality. The language, then, is seen within an interactionist perspective, not as an object, or code, but as a core element shared between the interlocutors and as an action of man on himself, others, the language itself and on reality; and
4. Discursive-humanist - which, akin to the previous approaches, centers on the subject and their linguistic manifestations within a social-interactionist context - and sees language as a shared object, but that is in constant interaction with the social, psychological and discursive systems, depending not only on the performance of the speaker, but on an entire social and dynamic construct, which is internalized by social structures and values that integrate and transform semiotic and social elements, then showing a contiguity with the enunciative formation, but with emphasis on subjective aspects and socio-cultural contexts, also analyzed by Pragmatics, Anthropology and Sociology.

It can be noted from this division, that these four areas give rise to different contributions in the field of Linguistics research and its relationship with Cognitive Neuropsychology, either reinforcing a more descriptive and classificatory theoretical approach, with the objective measurement of specific aspects of language functioning, or proposing other approaches and methodologies for interdisciplinary assessment of the cognitive processes underlying the comprehension and use of Language.

For a brief overview about these contributions in academic output related to aphasia and other acquired language disorders, refer to Table 1 which groups theses, dissertations and articles produced from 1980 to 2013 involving the fields of Linguistics, Aphasia and Language disorders under four different time periods. The research was based on the study of Carvalho^25^ and on:

**Table 1:** Academic output in Brazil in the field of Linguistics on Neurological Disorders Assessment.

	1980-1989			1990-2000			2001-2010			2011-2013	
	Dissertations and theses	Published manuscripts		Dissertations and theses	Published manuscripts		Dissertations and theses	Published manuscripts		Dissertations and theses	Published manuscripts
Organicist-localizationist	1	3		4	2		16	7		14	11
Neurolinguistic-structuralist	1	0		12	1		13	2		13	3
Enunciative-discursive	0	0		11	0		23	2		12	3
Discursive-humanist	0	0		4	2		14	3		10	2

(1) Theses repositories of CAPES;
(2) the Brazilian Library of Theses and Dissertations (BBTD);
(3) the Scientific Electronic Library Online database (Scielo); and
(4) the databases of dissertations and theses from leading universities that run graduate programs focused on this area, namely: USP, UNICAMP, PUC, UFRGS and UFRJ. For searching and sorting, the following keywords were used: "language", "aphasia", "language disorders" and "language evaluation". Titles, Abstracts and keyword terms were then analyzed to categorize each selected work under one of the four theoretical areas previously described.

Table 1 shows a marked increase in academic output from the 1990s when Linguistics began to have greater dissemination as evaluative research in the field of Language, becoming a more interrelated and multifaceted area in its relationship with the field of Cognitive Neuropsychology.

It is also worth noting the pattern of different trends for each period and how this matches with the lines of research in vogue. For example, with the consolidation of enunciative-discursive line in the 2001-2010 period and with greater equality of theoretical trends observed from 2011 onwards, perhaps for the wider dissemination of research interrelated with the fields of Neuroimaging and Cognitive Psychology, which certainly also affects the larger number of scientific articles related to the organicist-localizationist area.

Therefore, what can be noted in this analytical course is that Linguistics, in its multifaceted aspects, is present in a large number of studies discussing neuropsychological criteria related to the study of Aphasiology and dementias. The greatest contribution of Linguistics, however, is in proposing the interaction with the subject, analyzing its relationship with language [langue] and its practical and meaningful functioning.

There are many studies currently being structured, in which researchers are split between the theoretical construction and the orientation of studies under these methodological paradigms, in order to contribute to the possible investigative processes. These contributions offer gains but also challenges. On the one hand, it is necessary to investigate the linguistic phenomena in order to explain the standards of performance both in normal and injured brains. On the other hand, this investigation must be undertaken within the language's functional and effective routine use. That is, one cannot completely trust the result of a Language test and disregard the subject and their history. However, it is not feasible to conduct longitudinal studies in every piece of research. In other words, the investigative area needs suitable and adapted instruments – both neuropsychologically and culturally – and Linguistics should work according to this need, assisting in the preparation of these methodologies and enabling the devising of criteria and objective analytical and metalinguistic parameters that consider the individual, their history and constitution as a subject in and of the Language.

## PSYCHOLOGICAL CONTRIBUTION

**Cognitive Neuropsychology, Psycholinguistic and Psychometric Criteria in Language Assessment.** Not only Linguistics offered a framework for the evaluation of Language in brain lesion patients. Cognitive Psychology has also proposed hypothetical models of communication that include oral and written Language as well as different codification processing. In 1973, the seminar work of Marshall and Newcombe^26^ in England, introduced the cognitive approach in the evaluation of acquired written disorders. In Brazil, publication about cognitive processing of written Language appeared in the 1990s^27^ and the cognitive approach expanded to other Language modalities as well as to other cognitive function and their interaction with Language.

Concerned with how Language is performed by the individual, it considers environmental and non-environmental factors that affect psycholinguistic mechanisms: the role of psychological decoding and encoding structures that give meaning to words, sentences and texts. Cognitive Psychology encompasses these mechanisms in all forms of human communication, such as oral, written and gestural Language, in neuropsychological studies, particularly in the evaluation of patients with brain injuries. It also seeks to identify the influence of tasks and stimuli in psycholinguistic mechanisms and structures. The first studies in Cognitive Neuropsychology postulated the value of single case studies. Nevertheless, nowadays, along with Psychometrics, Cognitive Neuropsychology uses Psycholinguistic knowledge for the application of different forms of Language assessment.

Language can be examined directly through the use of linguistically represented verbal or visual stimuli. It may also be a mediator in the assessment of other cognitive processes, such as episodic memory, work and inhibition, among others. In both cases, the so-called "psycholinguistic criteria", such as familiarity, plausibility, level of abstraction, extension, among others, should be considered in the construction and final selection of instruction-stimuli sets. This is because these criteria may interfere in the duration or type of mental processing.

In the creation of an assessment instrument, items and Language tests should also be suitable for the verification of psychometric parameters, since these provide information about the quality of the standards (norms of reference, for instance), validity and reliability, in addition to sensitivity and specificity.^28,29^ In Neuropsychology, the parameters of sensitivity and specificity are essential because they are related to the diagnostic accuracy of a standardized instrument for detecting cognitive dysfunctions, i. e., they provide a basis for clinical decisions. Sensitivity is the accuracy of the test in correctly identifying a real cognitive alteration (true positives); complementary, specificity corresponds to the accuracy of the clinical tool in diagnosing the absence of cognitive impairment when it does not occur (true negatives).^30^

Such methodological concerns are essential to clinical activities since tools must measure what they are intended to assess and need to be adapted to the social, cognitive and communicative culture of the target population.^31-33^ Furthermore, psychometric evidence is needed to provide information both regarding the proper application of the test and for subsequent interpretation according to pre-established norms.^31^

A tripartite model has long been known as one of the ways to look for evidence of validity. It includes content validity (extent to which the content of the items is capable of measuring what is theoretically defined in the literature), criterion validity (extent to which the task is capable of predicting the performance of the individual being assessed), and construct validity (if what we are using really measures what is intended to be measured). The old validities are now being relabeled as types of evidence and divided into five main categories, namely:

(A) content-based evidence;
(B) evidence based on the response process;
(C) internal structure evidence;
(D) evidence based on the relationship with external variables; and
(E) evidence based on consequences of the assessment with a particular test.

In short, it is understood that the five category model of evidence uses the data obtained to analyze the construct inherent to the theoretical model that is being proposed/developed, even if the data obtained is not directly related and depend more on the interpretation of who is using them. Although this model is not new to the scientific environment internationally, in Brazil the tripartite model is still being used.

Furthermore, in psychometric analysis, in addition to the validities explored, studies of reliability and/or accuracy of the tests are being conducted. These are related to the consistency and repeatability of the measures when testing procedures are performed at different times in a population.^33^ To this end, it is possible to promote analysis of test-retest, reliability among raters and/or internal consistency. Lastly, evidence of validity of the scores of a test can be obtained through any systematic research that confirms or adds something to its meaning, regardless of when this occurs or who performs it.^33^ The interdisciplinarity of neuropsychological knowledge in the process of development of an instrument for its quantitative-qualitative interpretation is unquestionable.

## CURRENT PANORAMA

**Adaptation and creation of validated and standardized instruments.** Earlier Language assessment for brain lesion patients, in general, used techniques of analysis of oral discourse and tasks developed by the leaders of each training center. These assessments were based on a variety of theoretical approaches, which subsequently were disseminated in the clinical context by the professionals trained at the centers. Although they made clinical practice feasible, a number of problems arose from these practices, among them the use of new, adapted or translated instruments with no evaluation of their psychometric properties and without norms obtained in our population for their interpretation. Another problem was lack of access by professionals to new instruments developed following scientific advances in various sub domains of Language.

In this regard, recent research, especially in the last two decades, has focused on the examination of the psycholinguistic and psychometric properties and the validity of the instruments. Also there have been great advances in the adaptation of the psycholinguistic and cultural aspects of the tests and stimuli as well as in the construction of norms that can aid clinical practice. This evolution has promoted national and international multicenter studies.

In this section, after a search of the databases Pubmed, Scopus and Lilacs with the terms "aphasia", "language", "assessment", "Brazil", "dyslexia", "dysgraphia", "semantic memory", "naming" and "verbal fluency" in English and Portuguese, spanning the period from 2002 to July 2014, we have selected publications focusing on instruments for assessing Language and communication in adults already available or with the potential to be used in clinical practice. Since the terms were too general and led to an excessive number of entries retrieved in the databases, we needed to conduct a selection of tasks rather than a systematic review. Moreover, some instruments (e.g. verbal fluency task) were used in numerous studies with different purposes and it was beyond the scope of this review to include them all (the current focus is tools being studied or already available). Also, pure experimental tasks, language tests included in extended neuropsychological batteries or tests with no evidence of psycholinguistic control and/or psychometric data were excluded. The instruments were classified as:

1) Brief and expanded batteries for assessing Language/aphasia and communication;
2) Instrument for functional assessment of communication;
3) Expanded battery to assess linguistic subdomains;
4) Tasks to assess specific aspects of Language; and
5) Studies with norms on psycholinguistic stimuli (see references in Table 2).

**Table 2:** Brazilian panorama of instruments for Language Assessment

Authors	Year	Classification	Instrument	Availability for use in clinical practice
Radanovic & Mansur, Radanovic et al., Mansur et al.^38-41^	2002 2004 2005	Extended battery for language assessment/aphasia	Boston Diagnostic Aphasia Examination	
Ortiz et al.^37^	2011	Brief battery for language assessment/aphasia	M1-Alpha	X
Ishigaki et al.^36^	2013	Brief battery for language assessment/aphasia	MT Beta-86	X
Pagliarin et al.^34,35^	2014	Extended battery for language assessment/aphasia	Montreal-Toulouse Language Assessment Battery for aphasia	X
Neves et al.^42^	2014	Extended battery for language assessment/aphasia	Western Aphasia Battery	
Casarin et al.^45^	2014	Brief battery for language assessment/aphasia	Montreal Battery for Brief Communication Assessment	X
Fonseca et al.^43^	2008	Brief battery for communication assessment	Montreal Battery for Brief Communication Assessment	X
Carvalho et al.^46^	2008	Functional evaluation of language and communication (Interview)	ASHA- FACS	X
Carthery-Goulart et al.^65^	2013	Extended battery to evaluate a language subdomain	Cambridge Semantic Memory Battery (semantic memory evaluation)	
Mansur et al.^67^	2013	Instrument to evaluate a specific skill	Pyramid and Palm Trees test/ Kissing and Dancing Test - Noun and Verb Semantic Association Tests	
Mansur et al.^58^ Miotto et al.^59^	2006 2010	Instrument to evaluate a specific skill	Boston Naming Test (noun naming)	
Paula et al.^62^ Carvalho et al.^61^	2012 2009	Instrument to evaluate a specific skill	Token Test - Auditory comprehension of sentences (commands)	
Rodrigues et al.^64^	2013	Instrument to evaluate a specific skill	Word and Nonword Writing task	
Spezzano et al.^60^	2013	Instrument to evaluate a specific skill	Verb and Object Naming Battery (verb and noun naming)	
Brucki & Rocha^47^	2004	Instrument to evaluate a specific skill	Category Fluency (animals/min)	X
Radanovic et al.^48,55^	2007 2009	Instrument to evaluate a specific skill	Category Fluency (animals/min) and (fruit/min)	X
Caramelli et al.^49^ Fichman et al.^50^ Passos et al.^51^ Araujo et al. ^52^ Jacinto et al. ^56^ Bertola et al.^57^	2007 2009 2011 2014 2014 2014	Instrument to evaluate a specific skill	Category Fluency (animals/min)	X
Senhorini et al.^53^	2006	Instrument to evaluate a specific skill	Letter Fluency (establish letters F,A,S as more appropriate for letter fluency in Portuguese)	X
Machado et al.^54^	2009	Instrument to evaluate a specific skill	Letter Fluency (letters F,A,S)	X
Pompéia et al.^77^	1998	Psycholinguistic criteria for stimuli	Pictures to be used in research	X
Sardinha^78^	2003	Psycholinguistic criteria for stimuli	Frequency norms for words in Portuguese	X
Janczura et al.^79^	2007	Psycholinguistic criteria for stimuli	Imageability norms for nouns in Brazilian Portuguese	X

As can be seen regarding the batteries for Language assessment and diagnosis of aphasia (profile and severity), there is the Montreal-Toulouse protocol, in brief and expanded versions.^34-37^ This protocol has undergone the steps of psycholinguistic and cultural adaptation of linguistic and pictorial items, evaluation of psychometric properties/diagnostic validity and standardization, and is soon to be marketed in Brazil (MTL Brasil, Parente et al., manuscript not published). This instrument includes tasks assessing automatic language, auditory and written comprehension, oral and written speech (discourse), writing to dictation and copy, oral repetition, reading, verbal fluency, naming, calculus, object manipulation, non-verbal praxis and arithmetic skills. There are also norms with cross-cultural adaptation of the Boston test for assessment of aphasia^38-41^. The protocol however, is available only for research. Finally, we have identified a study with the Western Battery for assessment of aphasia that is in a very early stage of adaptation for use in our environment (linguistic and conceptual parameters).^42^

In another important category, is the work to adapt the MAC battery for communication assessment,^43^ an adapted version of the Protocole MEC^44^. This battery goes beyond the formal aspects of Language (phonological, lexical-semantic and morphosyntactic levels) and investigates supra-segmental and pragmatic aspects, such as comprehension and prosody production, as well as comprehension of figurative Language and speech. It has norms for the adult population and may also be purchased commercially for clinical use. Recently, the brief version of the test, MAC B, which evaluates the same cognitive processes as MAC plus writing and reading skills in a third of the time, was made available for academic and clinical purposes.^45^ Studies have already been conducted with this battery in patients with traumatic brain injury and with right and left brain focal lesions.

For functional assessment of communication, the ASHA-FACS questionnaire^46^ has standards for use with Alzheimer's disease patients. Applied to relatives of patients with Language and communication difficulties, it seeks to identify the functional impact of communication changes on daily activities. This instrument is extremely important for planning interventions and its use in other clinical groups should be investigated.

Regarding tests that assess specific aspects, we highlight the verbal fluency tasks, which assess the interaction between lexical and semantic access and executive functions. They are relatively sensitive for detecting Language disorders and are present in numerous batteries for screening cognitive decline.^47-57^ Among the naming tests, the most widely used in our milieu in the context of research is the American version of the Boston test, with the standards published by Mansur et al. (2006).^58^ Recently, norms for the items of this test and a proposed cultural adaptation^59^ have been published; these proposed the replacement of 20 items considered inappropriate for our context, with reference values according to schooling. Reference values were recently published for a task that involves not only object but also verb naming.^60^ For auditory comprehension, the Token test has been translated^61^ and applied to elderly and patients with dementia^62^ with norms being provided for this population and the influence of schooling verified. Reference values for the elderly were also studied for the Test of Reception of Grammar (TROG-2) that provides a more detailed assessment of morphosyntactic aspects of sentence comprehension.^63^ In addition, there is a task for the evaluation of writing of words and pseudowords^64^ devised according to Psycholinguistic and Cognitive Neuropsychology criteria. A version of this task was created to assess reading (Rodrigues and Salles, manuscript submitted). In initial phases of research, instruments are available focusing on the assessment of semantic memory, such as the Cambridge semantic memory battery ^65^ used in patients with Primary Progressive Aphasia in Brazil^66^ and the tests of semantic association Pyramids and Palm Trees, and Kissing and Dancing^67^ instruments, have been investigated as relevant tools for the diagnosis of neurodegenerative syndromes such as primary progressive aphasia, particularly the semantic variant.^68,69^

Although short or expanded neuropsychological batteries were not selected for this survey, we highlight, in the context of Language assessment, the NEUPSILIN battery^70-74^ for the extensive work on the psycholinguistic adequacy of its constituent items and its specially developed version for non-fluent aphasics, the NEUPSILIN-Af ^74^ which represents a significant differential. Among the short batteries, we emphasize the ACE-R, which has a somewhat more comprehensive Language assessment than other screening tests and has undergone the steps of psychometric adaptation to our environment.^75-76^

Finally, we should highlight the initiatives for characterization of linguistic stimuli that do not consist of assessment materials in themselves, but which are essential for the construction of assessment instruments for clinical practice and research.^77-80^ Due to these studies, therapists and researchers can draw on norms of frequency, imageability and semantic association of words, as well as pictures whose familiarity, naming agreement and visual complexity have been evaluated.

In short, there is a promising scenario for Language assessment in Brazil, with expectation in the near future of an increase in the number of instruments available for clinical practice and research in this area.

## FINAL REMARKS

In this historical review we have focused on more than 40 years of evolution in the Language assessment of brain damaged patients in Brazil. Multidisciplinary knowledge has enriched methods of evaluation and increased the diversity of approaches. Some Language batteries have been constructed recently after concerted efforts of validation and normatization for our culture. Nevertheless, it is noteworthy that a good Language evaluation does not depend on the quality of the tool alone, but relies heavily on the interpretation of its results. For this task, theoretical knowledge is required.

We have also noted that, during this period, the Language assessment that hitherto focused on aphasia, is now directed toward other acquired neurological diseases, such as dementia. Moreover, Language is not considered an isolated function; the relationship between Language disorders and other cognitive functions are taken into account in patient evaluation.

There is much more to be done in terms of Language evaluation for the coming decades. While only a few tests and batteries are available in the Portuguese language, instruments must keep pace with advances in scientific knowledge, and new instruments will be required. In this aspect, specific tasks must be constructed to test Language aspects during neural imaging, and thus promote a finer integration of Language and its neural organization. Moreover, we cannot neglect the introduction of computational analyses, which can help in diagnoses and provide a sophisticated view of the patient's Language failures.

Despite the stark contrast between the 1970s evaluations and practices during the decade of 2010, the coexistence of two different focuses in evaluating brain lesion patients indicated in the earlier period continues. It is valuable to know how Language is disturbed or preserved after a brain lesion, and also how the patient in his environment and social parameters react to a new situation. Norms are useful for diagnosis and for multicenter collaborative studies, but cannot substitute the richness of case studies that emphasize the diversity and the singularity of the effect of a brain lesion on Language and on the communication of a given patient.

## Footnotes

* **Note**. There is a difference between *language* and *human* speech or the *human faculty of language*. In this paper, **Language** is defined as the human capacity for acquiring and using complex systems of communication, and a **language** is any specific example of such a system.
